# Quantitative evaluation of acid flow behavior in fractures and optimization of design parameters based on acid wormhole filtration losses

**DOI:** 10.1038/s41598-024-66680-z

**Published:** 2024-07-09

**Authors:** Sen Yang, Kaige Zheng, Jian Zhang, Nan Dai, Lintao Wang, Zeyang Wang, Haojie Wang, Xiangwei Kong

**Affiliations:** 1 Xi’an Research Institute of China Coal Technology & Engineering Group Corp, Xi’an, 710077 China; 2 School of Petroleum Engineering, Yangtze University, Wuhan, 430100 China

**Keywords:** Carbonate, Acidizing, Flow model, Acid etching wormhole, Acid filtration, Acidizing dynamic simulation, Energy science and technology, Engineering

## Abstract

The technique of matrix acidification or acid fracturing is commonly utilized to establish communication with natural fractures during reservoir reconstruction. However, this process often encounters limitations due to filtration, which restricts the expansion of the primary acid-etching fracture. To address this issue, a computational model has been developed to simulate the expansion of an acid-etching wormhole by considering various factors such as formation process, injection duration, pressure build-up, and time-varying acid percolation rate. By analyzing the pumping displacement of acid-etching wormholes, this model provides valuable insights into the time-dependent quantities of acid percolation. It has been revealed that the filtration rate of acid-etching wormholes is strongly influenced by pumping displacement, viscosity, and concentration of the acid fluid used in stimulation as well as physical properties of the reservoir itself. Notably, viscosity plays a significant role in determining the effectiveness of acid fracturing especially in low-viscosity conditions. Acid concentration within 15% to 20% exhibits maximum impact on successful acid fracturing while concentrations below 15% or above 20% show no obvious effect. Furthermore, it was found that pumping displacement has a major influence on effective fracturing. However, beyond a certain threshold (> 5.0 m^3^/min), increased pumping displacement leads to slower etching distance for acids used in construction purposes. The simulation also provides real-time distribution analysis for acidity levels within eroded fractures during matrix-acidification processes and quantifies extent of chemical reactions between acids and rocks within these fractures thereby facilitating optimization efforts for design parameters related to matrix-acidification.

## Introduction

The global advancement of oil and gas field development has reached its intermediate to advanced stages, raising concerns about the depletion of subsurface resources and prolonged decline in reservoir pressure during extraction. Consequently, the stability of existing production areas is being challenged by an increasing number of low-pressure oil and gas wells that have undergone various damaging operations such as drilling completion activities, acid fracturing workovers, well killing, among others. These operations lead to issues such as scale imbalance, sludge accumulation, bacterial growth, precipitation build-up, particle migration, as well as external operational interventions^1,2^. It is essential to minimize formation damage for gas wells, and acidification procedures play a crucial role in achieving this objective by mitigating or eliminating damage while enhancing connectivity between nearby oil and gas conduits at the well sites^3–6^. Technicians recommend frequent use of foam acid systems with restricted fluid volumes that possess robust suspension capabilities, facilitating easy flowback during repetitive acidification processes aimed at addressing substantial formation damages within their proximity.

Fracturing and acidizing techniques are indispensable for the construction, stabilization, and enhancement of oil and gas deposits in carbonate formations. These methods play a crucial role in exploration, reserve verification, production increase, recovery enhancement, as well as cost-effective and efficient development of oil and gas fields. The principles underlying fracture stimulation and acidification revolve around three key aspects^7–13^. The creation of artificial fractures serves to expand the seepage area for oil and gas to flow into the wellbore. Additionally, it aids in eliminating contamination surrounding the wellbore. Lastly, it facilitates communication between high-permeability zones, fractures, and hydrocarbon-bearing zones within the reservoir. Therefore, evaluating the effectiveness of acid fracturing primarily relies on assessing both effective distances covered by acid etching as well as its conductivity.

In carbonate reservoirs, natural fractures and karst caves are commonly encountered, resulting in significant heterogeneity. The fracture-cavity system serves as the primary medium for oil and gas accumulation and seepage, while the matrix lacks sufficient reservoir capacity. To achieve effective acid etching fracture formation through acid fracturing, it is essential to ensure optimal communication and connectivity within the fracture network to enhance stimulation effects^14–19^. However, the occurrence of the wormhole effect during acid fracturing significantly restricts the extension of the main acid etch fracture, leading to suboptimal or even ineffective stimulation in numerous oil and gas wells. Fracture conductivity depends on both the extent of rock dissolution by acidic solution and its etching pattern. To attain high fracture conductivity, adequate dissolution of rock minerals must be achieved through a reaction between acidic solution and fracture walls; however, this process may not result in uniform etching^20–24^. The formation of wormholes increases fluid filtration rate which reduces effective rock dissolution by acidic fluids along with narrowing down fractured width due to accelerated local acidity-rock reactions on artificial fracture walls. These combined factors fall short in achieving desired stimulation effects compared to increasing length of far-well region's connected-acid—etching fractures.

Hoefner et al.^25^ employed a network model to illustrate the flow of acid in pores and simulated pore flow and reaction by representing the pore medium through interconnected two-dimensional network nodes connected by cylinders. This model improved qualitative predictions of dissolution, mass transfer limitation, or surface reaction limitation. Daccord et al.^26^ proposed utilizing fractal dimension to characterize acid wormholes in limestone acidizing, which captures certain features of etching holes but struggles to fully capture their geometric intricacies. Hill^27^ calculated the comprehensive filtration coefficient of an acidic solution with wormholes based on assumptions from classical filtration theory that neglect pressure drops within the wormhole and assume linear acid flow in invaded areas; however, as a wormhole develops, acid liquor enters formations via filtration from both its tip and wall along its length and width. Bazin et al.^28^ study demonstrated phenomenology during the process of acid fracturing while proposing an experimental approach that includes measuring wormhole propagation velocities, leak-off volumes, observing dissolution patterns, and evaluating various representative conditions for fluid performance during acid flow into fractures. Mou et al.^29^ proposed an innovative model to simulate the acid leak-off phenomenon in naturally fractured carbonate oil reservoirs during acid fracturing. This model incorporates the chemical reaction between acid and rock, considers the variation in fracture width caused by rock dissolution on fractured surfaces, and accounts for fluid flow within naturally fractured carbonate oil reservoirs. Zhu et al.^30^ conducted an extensive study to investigate the influence of various acid types combined with carbonate on fracture morphology at different temperatures, along with examining the viscosity relationship for each fluid combination. The authors thoroughly examined and analyzed the variations in etched fracture wall morphology among these seven liquid combinations. Additionally, they simulated the conductivity of acid—etching fractures under varying closure stress levels. Katende et al.^31^ employed a multi-scale approach to investigate proppant embedment and fracture conductivity, ranging from nano-scale instrumented micro/nano-indentation to millimeter (mm) scale mono-layer propped fracture flow at reservoir temperature and pressure, as well as American Petroleum Institute (API)-RP19D conductivity tests using inch/cm-scale shale platens.

Farrokhrouz et al.^32^ investigated the acidizing of propped fractures by differing experimental parameters to optimize the fracture conductivity through acidizing, which determined the optimal fracturing design parameters (HCl concentration, proppant concentration, proppant size and acid injection rate) for hydraulic fracturing in Eagle Ford shale. The effects of proppant and its embedded fracture wall on fracture conductivity were studied by experimental and mathematical models. The effects of rock mineralogy, surface roughness, fluid, confining pressure, time, temperature and bedding on proppant embedment were discussed more thoroughly by Katende et al.^33–36^. Ameri et al.^37^ conducted simulations using fracturing software to study acid fracturing treatments for a tight limestone reservoir within a shale formation. Their research focused on investigating the optimal design parameters for acid fracturing in order to achieve longer and wider fractures with higher conductivity. Jia et al.^38^ employed a novel dual-scale continuous medium model to investigate the two-dimensional acidification process, and substituted the Darcy equation with the Navier–Stokes–Darcy equation to describe the flow behavior of acid in a fracture-pore dual medium. Liu et al.^39^ integrated a model for calculating real-time acid-rock reaction enthalpy into heat transfer and fracture growth models, thereby simulating its impact on the dimensions of etched fractures. Xue et al.^40^ performed leak-off experiments under high temperature and high-pressure conditions to investigate the leak-off behavior in matrix, wormhole, and fracture respectively. They developed a wormhole leak-off model that considers both matrix and fracture medium. Katene et al.^41^ identifies and describes important processes occurring during proppant embedment, hydraulic fracturing, laboratory testing of fracture conductivity, as well as modeling of proppant embedment. Wang et al.^42^ established an expansion model for gelling acid wormholes under radial conditions, enabling simulation of their expansion characteristics. This research optimized construction parameters and provided the basis for optimal design of carbonate reservoir matrix acidizing. Aljawad et al.^43^ proposed a comprehensive approach to enhance understanding of acid fracturing through field observations, laboratory experiments, and modeling perspectives. Their work aimed at bridging the gap between job execution outcomes and predictions made by acid fracture models. Tardy et al.^44^ established the mathematical models of particle transport, bridging, blocking, diversion and degradation in the acidification process, which could introduce and describe the matrix acidification process in detail.

Although extensive research has been conducted on acid-erosion wormholes and numerous experiments have been carried out to investigate acid fluid filtration loss, there remains a lack of systematic discussion on the morphological characteristics and quantitative calculation methods of these wormholes. Therefore, it is crucial to consider the acid fluid loss mechanism of acid-erosion wormholes and devise effective strategies for controlling such filtration loss in order to enhance the efficiency of carbonate rock acidizing. A 3D fracture expansion model was developed to simulate the dynamic propagation of fractures during acid fracturing operations. This model integrates the effects of fluid pressure drop along the vertical direction of the fracture, fluid gravity, geostrophic gradient, and geostrophic differential. Furthermore, a 3D mathematical model of acid flow reactions in the acid—etching fracture was established to analyze both longitudinal mass transfer and vertical acid flow in the fracture.

## Simulation of acid flowing and reaction in the three-dimensional acid etching fracture

### Mathematical model of acid flowing in the three-dimensional acid etching fracture

**Figure 1:**
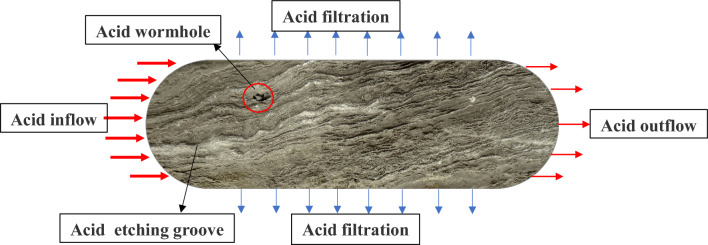
Schematic diagram of acid flow etching inside fracture.

Acid flows through the fracture, causing etching of the fracture wall and subsequent loss through both matrix and pore filtration, as illustrated in Fig. 1. The process of acid flow within the fracture can be accurately described using a mathematical model. The three-dimensional flow reaction mathematical model of acid incorporates the migration of acid along the length and height of the fracture, as well as convection and diffusion in the direction of fracture width and height.

Model assumptions are as follows. The surface reaction rate governs the reaction process between acid solution and reservoir rock. The acid-rock reaction only occurs in the area of the rock with a very thin fracture wall. The acid-rock reaction area in the filtration zone exhibits single-phase seepage. The filtration zone contains only the liquid acid solution and the solid rock. The carbonate minerals exhibit a preferential reaction with HCl. The solid rock and the acid solution are both considered to be incompressible. The impact of gravitational forces on the fluid is disregarded.

The continuity equation of the model is as follows:

1 $$\frac{\partial \left(C{v}_{x}\right)}{\partial x}+\frac{\partial \left(C{v}_{y}\right)}{\partial y}+\frac{\partial \left(C{v}_{z}\right)}{\partial z}=\frac{\partial }{\partial y}\left({D}_{e}\frac{\partial C}{\partial y}\right)+\frac{\partial }{\partial z}\left({D}_{e}\frac{\partial C}{\partial z}\right)$$

Boundary conditions:

$$  \left\{ {\begin{array}{*{20}l}    {x = 0\;C = C_{{inj}} } \hfill  \\    {y = 0\frac{{\partial C}}{{\partial y}} = 0} \hfill  \\    {y =  \pm \frac{W}{2}\left( {C_{l}  - C_{B} } \right)v_{l}  + D_{e} \frac{{\partial C}}{{\partial y}} + K\left( {1 - \varphi } \right)\left( {C_{B}  - C_{{eq}} } \right)^{m}  = 0} \hfill  \\   \end{array} } \right. $$

where $C$ is acid concentration, mol/L. *ν* is the flow rate of acid, m/s. ${D}_{e}$ is effective mass transfer coefficient. *m* is reaction order. *k* is the constant of reaction rate, m/s. *C*_*B*_ is acid concentration at fracture wall, mol/L. *C*_*l*_ is filtration loss acid concentration, mol/L. *C*_*eq*_ is the equilibrium of acid concentration, mol/L. *v*_*l*_ is filtration rate, m/s.

### The three-dimensional acid flowing field

**Figure 2:**
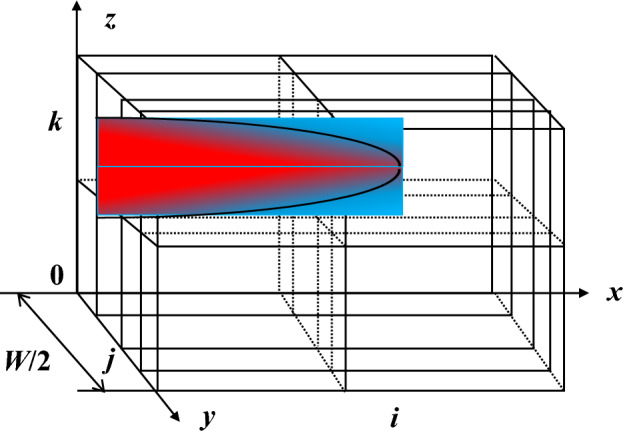
Schematic representation of the spatial distribution of acid concentration in a 3D fracture.

To solve Eq. (1), it is necessary to first calculate the acid flow rate at each point of the fracture. By utilizing the existing velocity field grid, our focus will be on researching the half width of the fracture and subsequently dividing the fracture grid along its width direction. This approach will result in obtaining a three—dimensional differential grid, as depicted in Fig. 2.

*V*_*x*_ and *V*_*z*_ calculated from the previous velocity field simulation are the average velocity of acid fluid along the direction of fracture length and fracture height without considering the influence of fracture wall. The actual velocity changes along the width of the fracture, and the three-dimensional velocity field distribution of the acid liquid is calculated according to the fluid velocity distribution in the flat laminar flow. When the acid flow behaves as a power-law fluid (${n}^{\prime}$ is the flow regime index), the partial velocities of a grid acid in the direction of fracture length, fracture height and fracture width are:

2 $${v}_{x}=\frac{2{n}^{\prime}+1}{{n}^{\prime}+1}{V}_{x}\left(1-{\left(\frac{2y}{W}\right)}^{\frac{{n}^{\prime}+1}{{n}^{\prime}}}\right)$$

3 $${v}_{z}=\frac{2{n}^{\prime}+1}{{n}^{\prime}+1}{V}_{z}\left(1-{\left(\frac{2y}{W}\right)}^{\frac{{n}^{\prime}+1}{{n}^{\prime}}}\right)$$

4 $${v}_{y}={V}_{L}\frac{2{n}^{\prime}+1}{{n}^{\prime}+1}\left(\left(\frac{2y}{W}\right)-\frac{{n}^{\prime}}{2{n}^{\prime}+1}{\left(\frac{2y}{W}\right)}^{\frac{2{n}^{\prime}+1}{{n}^{\prime}}}\right)$$

When the acid solution exhibits Newtonian fluid behavior, the partial velocities of the grid acid solution in the directions of fracture length, fracture height, and fracture width are respectively:

5 $${v}_{x}=\frac{3}{2}{V}_{x}\left(1-{\left(\frac{2y}{W}\right)}^{2}\right)$$

6 $${v}_{z}=\frac{3}{2}{V}_{z}\left(1-{\left(\frac{2y}{W}\right)}^{2}\right)$$

7 $${v}_{y}={V}_{L}\left(\frac{3}{2}\left(\frac{2y}{W}\right)-\frac{1}{2}{\left(\frac{2y}{W}\right)}^{3}\right)$$

### Difference equation of acid-rock reaction and its solution

The Eq. (1) is discretized using the central difference scheme.

8 $${v}_{x}\frac{{C}_{i,j,k}-{C}_{i-1,j,k}}{\Delta x}+{v}_{y}\frac{{C}_{i,j+1,k}-{C}_{i,j-1,k}}{2\Delta y}+{v}_{z}\frac{{C}_{i,j,k+1}-{C}_{i,j,k-1}}{2\Delta z}=\frac{1}{\Delta {y}^{2}}{{D}_{e}}_{i-1,j,k}\left({C}_{i,j+1,k}-{C}_{i,j,k}\right)-\frac{1}{\Delta {y}^{2}}{{D}_{e}}_{i-1,j-1,k}\left({C}_{i,j,k}-{C}_{i,j-1,k}\right)+\frac{1}{\Delta {z}^{2}}{{D}_{e}}_{i-1,j,k}\left({C}_{i,j,k+1}-{C}_{i,j,k}\right)-\frac{1}{\Delta {z}^{2}}{{D}_{e}}_{i-1,j,k-1}\left({C}_{i,j,k}-{C}_{i,j,k-1}\right)$$

9 $$-{D}_{ei-1,Ny,k}\frac{{C}_{i,Ny,k}-{C}_{i,Ny-1,k}}{\Delta y}=-k\left(1-\phi \right){C}_{i-1,Ny,k}^{m}$$

It is sorted out,

10 $$a{a}_{i,j,k}{C}_{i,j,k-1}+b{b}_{i,j,k}{C}_{i,j-1,k}+c{c}_{i,j,k}{C}_{i,j,k}+d{d}_{i,j,k}{C}_{i,j+1,k}+e{e}_{i,j,k}{C}_{i,j,k+1}=g{g}_{i,j,k}$$

where $$a{a}_{i,j,k}=\frac{{v}_{z}}{2\Delta z}+\frac{1}{\Delta {z}^{2}}{D}_{ei-1,j,k-1}$$, $$b{b}_{i,j,k}=\frac{{v}_{y}}{2\Delta y}+\frac{1}{\Delta {y}^{2}}{D}_{ei-1,j-1,k}$$, $$e{e}_{i,j,k}=-\frac{{v}_{z}}{2\Delta z}-\frac{1}{\Delta {z}^{2}}{D}_{ei-1,j,k}$$, $$g{g}_{i,j,k}=-\frac{{v}_{x}}{\Delta x}{C}_{i-1,j,k}$$, $$c{c}_{i,j,k}=-\frac{{v}_{x}}{\Delta x}-\frac{1}{\Delta {y}^{2}}{D}_{ei-1,j,k}+\frac{1}{\Delta {y}^{2}}{D}_{ei-1,j-1,k}-\frac{1}{\Delta {z}^{2}}{D}_{ei-1,j,k}\frac{1}{\Delta {z}^{2}}{D}_{ei-1,j,k-1}$$, $$d{d}_{i,j,k}=-\frac{{v}_{y}}{2\Delta y}+\frac{1}{\Delta {y}^{2}}{D}_{ei-1,j,k}$$

The fracture wall boundary conditions are processed as follows,

11 $${C}_{i,Ny,k}={C}_{i,Ny-1,k}-\frac{\Delta y}{{D}_{ei-1,j,k}}k\left(1-\phi \right){C}_{i-1,Ny,k}^{m}$$

Treatment of boundary conditions of fracture center:

Where $$j=1$$

12 $$ C_{i,0,k} = C_{i,j,k} \;bb_{i,j,k} = 0 $$

13 $$ cc_{i,j,k} = cc_{i,j,k} + \frac{{v_{y} }}{2\Delta y} + \frac{{D_{ei - 1,j,k} }}{{\Delta y^{2} }} $$

Equation (10) yields a five-diagonal equation system for the acid concentration, with an additional boundary condition. The distribution of acid concentration can be solved using the strong implicit method. Based on this, the acid concentration distribution for the next fracture section can be sequentially determined.

## Analysis of simulation results

Through the utilization of a solution method for filtration calculation, this study integrates models of acid fracture construction with features such as acid-etched wormholes. The primary objective is to assess the impact of these wormholes on construction and provide precise parameters for determining optimal choices regarding acidic substances, viscosity properties, consumption, and discharge capacities during site optimization procedures. These comprehensive parameters encompass various aspects including fluid filtration within acidic substances used in conjunction with their effects on wormhole development status and dynamic geometry within fractured areas subjected to etching processes. Furthermore, they can be employed to ascertain whether additional process measures are necessary to avoid blindly selecting parameters for acid fracturing construction and maximize the success rate.

### Influence of wormhole filtration on acid fracturing measures

**Figure 3:**
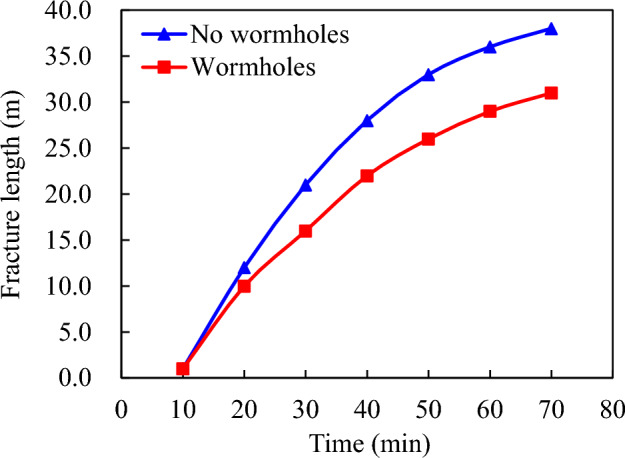
Impact of acid etching wormhole on the interface between acid and liquid.

The prepad fluid has a viscosity of 120 mPa s, a pumping displacement rate of 3.0 m^3^/min, and a liquid volume of 100.0 m^3^. The acid solution has a viscosity of 15 mPa s, a pumping displacement rate of 4.0 m^3^/min, and a liquid volume of 160.0 m^3^. The natural fracture density in the formation is estimated at 40 pieces/meter with fracture widths ranging from approximately 10 to 200 μm. The acid concentration is at 20%. As shown in Fig. 3, it is apparent that the magnitude of acid fluid filtration significantly impacts dynamic fracture width during the process of acid etching construction; without considering acid wormhole filtration, the filtrate volume at the end of construction measures at 23.0 m^3^.

**Figure 4:**
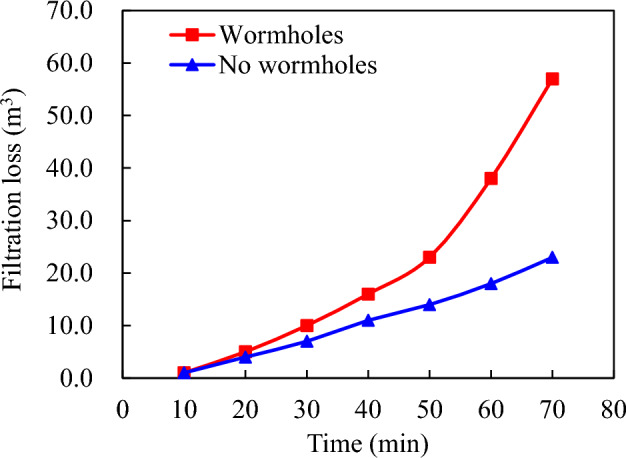
Influence of acid—etching worm holes on acid fluid filtration loss.

**Figure 5:**
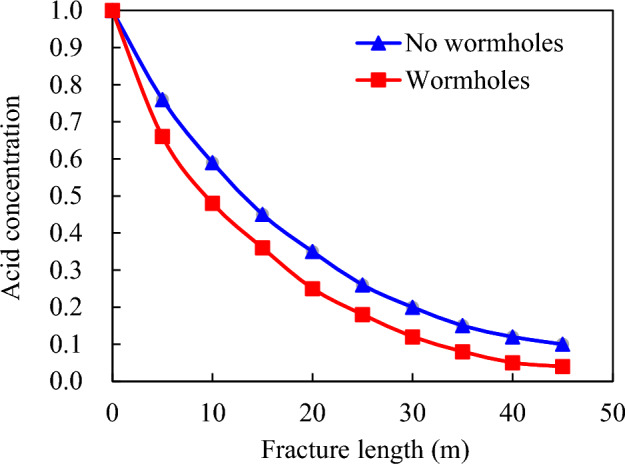
Influence of acid—etching worm hole on acid concentration distribution in fracture.

However, the formation of wormholes increases this value to 57.0 m^3^, indicating a significant enhancement in acid liquor filtration (Fig. 4). The presence of acid wormholes significantly reduces the effective distance for acid etching due to improved filtration effects. The data presented in Fig. 5 indicates that the effective acid etching distance is 44.5 m without wormhole filtration, whereas accounting for wormhole filtration results in a significant decrease to 31.0 m.

### Influence of acid fracturing parameters on acid etching wormhole filtration

#### Viscosity

**Figure 6:**
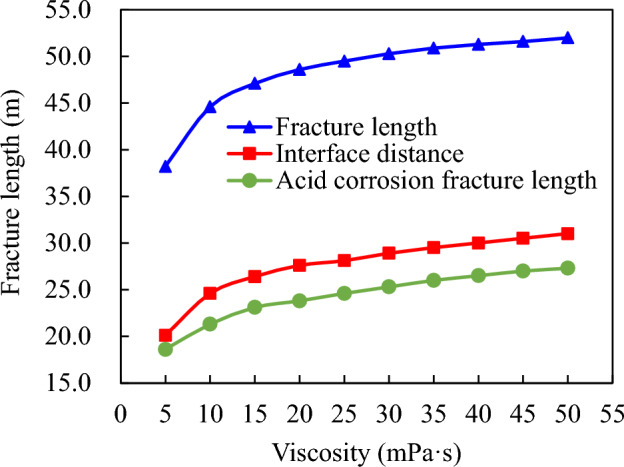
Influence of acid viscosity on acid fracture parameters.

**Figure 7:**
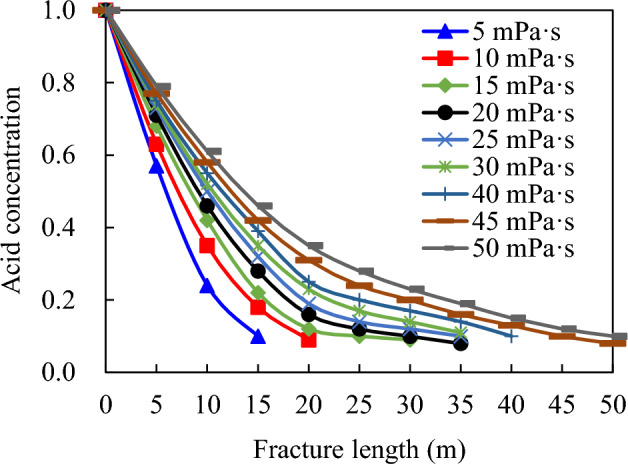
Influence of acid viscosity on acid concentration distribution in acid fracture.

The acid concentration is 20%, with an acid consumption of 160.0 m^3^ and a pumping displacement of 4.0 m^3^/min. The calculated viscosity for comparison ranges from 5 to 50 mPa s. Figure 6 illustrates a significant increase in the total length of acid fracturing fracture, acid interface, and effective distance of acid etching as viscosity increases within the range of low viscosities (1–5 mPa s). However, within the range of viscosities between 5 and  20 mPa s, these parameters exhibit slower growth rates with increasing viscosity. Once exceeding a threshold value of approximately > 20 mPa s (Fig. 7), they become less sensitive to changes in acid viscosity.

**Figure 8:**
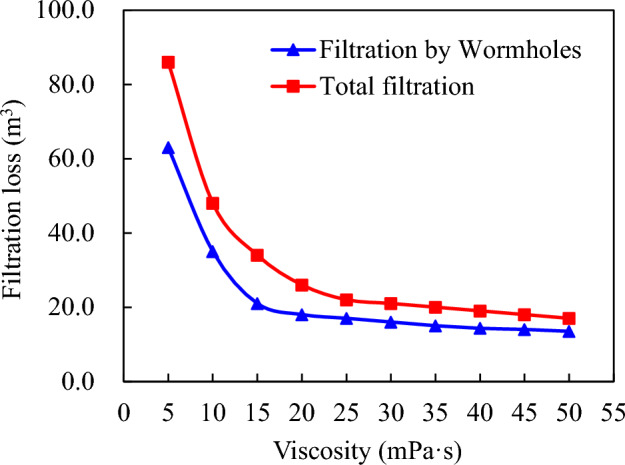
Influence of acid viscosity on acid filtration loss in acid fracture.

Due to significant filtration loss of acidic solution into natural fractures or matrix pores at lower viscosities (< 5 mPa s), intensified filtration occurs due to reactions between the acid solution and rock surfaces, leading to expansion of natural fractures and pores caused by wormholes. As shown in Fig. 8, at a viscosity of only < 5 mPa s, the total leaked amount reaches up to approximately 88.0 m^3^ with wormhole-induced filtration accounting for about 62.0 m^3^. However, the increase in viscosity effectively controls this filtration process. Once it exceeds 25 mPa s, acidic filtration becomes essentially controlled, reducing wormhole-induced filtration down to around 5.0 m^3^. This phenomenon can be further observed from the distribution curve depicting acidity concentrations within fractures after completion of acid fracturing.

#### Acid concentration

**Figure 9:**
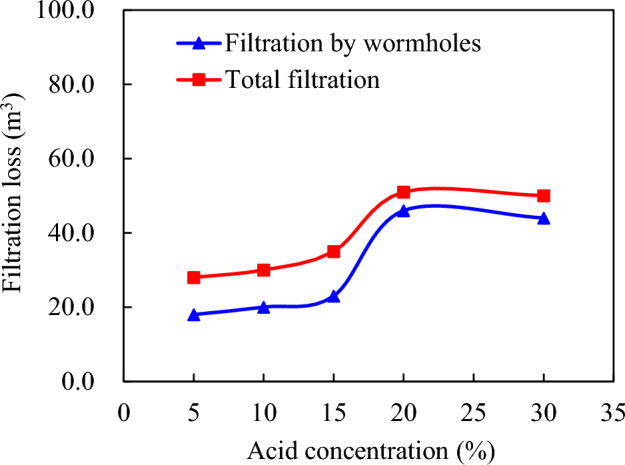
Influence of acid concentration on acid filtration in acid fracture.

Acid viscosity 20 mPa·s, pumping displacement 4.0 m^3^/min, acid consumption 160.0 m^3^. Acid concentration: 5%, 10%, 15%, 20%, 25%, 30%. Figure 9 demonstrates that when the concentration of an acid solution is relatively low (10% to 15%), the variation in the reaction rate of acid-rock interaction is not conspicuous. This is because within this specific range, the difference in the expansion velocity of acid—etching wormholes is not pronounced, and the acid concentration has a minimal impact on filtration. However, as the concentration of the acid solution increases from 15 to 20%, the acid—rock reaction accelerates, accompanied by an escalation in the expansion of acid—etching wormholes. This acceleration is characterized by a steep rise in filtration. For instance, when the acid concentration reaches 20%, the filtration loss induced by acid—etching wormholes is nearly double that of a 15% acid concentration.

**Figure 10:**
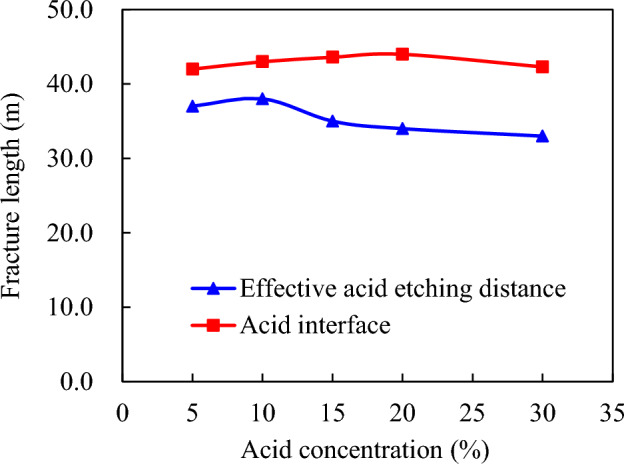
Acid concentration versus interface and effective acid etching distance.

Furthermore, when the acid concentration surpasses 25%, a phenomenon occurs where the acid-rock reaction does not necessarily gain momentum with the increase in acid concentration, but rather experiences a slight decline. It should be noted that an excessive amount of acid liquor filtration hinders the movement of the acid liquor interface and the ultimate effective distance of acid etching, as illustrated in Fig. 10. This highlights the importance of maintaining an optimal acid concentration to ensure efficient acid etching with minimal filtration loss.

#### Acid injection displacement

**Figure 11:**
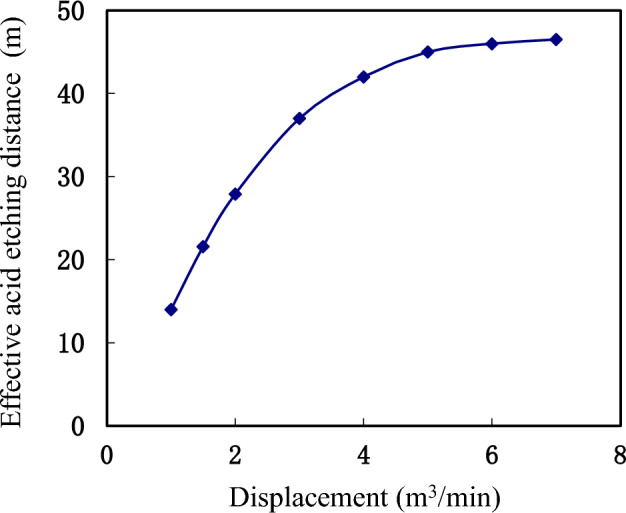
Influence of pumping displacement on effective action distance of acid solution.

Acid viscosity 10 mPa·s, acid concentration 20%, acid consumption 160.0 m^3^. Pumping displacement: 1.0, 2.0, 3.0, 4.0, 5.0, 6.0 and 7.0 m^3^/min. The impact of acid pumping displacement on the ultimate effective acid etching distance is a complex process that exhibits a linear increase in etching distance with increasing pumping displacement between 1.0 and 7.0 m^3^/min. This phenomenon is primarily attributed to the proportional increase in displaced acid volume as pumping displacement increases, resulting in greater etching distances. For instance, Fig. 11 illustrates how the penetration distance at 5.0 m^3^/min is nearly four times that at 1.0 m^3^/min. However, beyond 5.0 m^3^/min, this linear relationship becomes less apparent due to the progressively slower expansion of wormholes corroded by acid.

**Figure 12:**
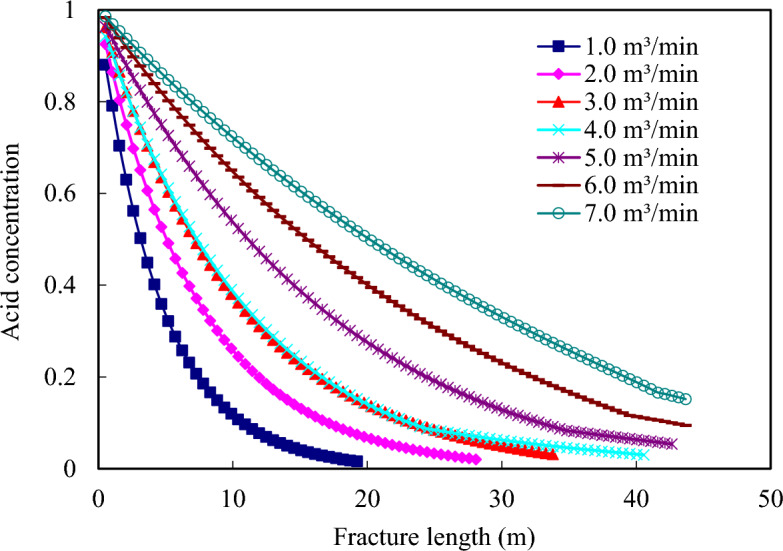
Influence of pumping displacement on acid concentration distribution in acid fracture.

These wormholes are formed and expanded due to the acid liquor seeping into the fractures or matrix of the rock. The acid rock reaction within the wormholes is slowed down due to a combination of factors, such as the ionic effect and the diffusion of acid concentration within the acid etching pores (Fig. 12). This diffusion process is unable to keep pace with the rapid expansion of the acid etching wormholes, resulting in a deceleration of the overall acid etching action.

**Figure 13:**
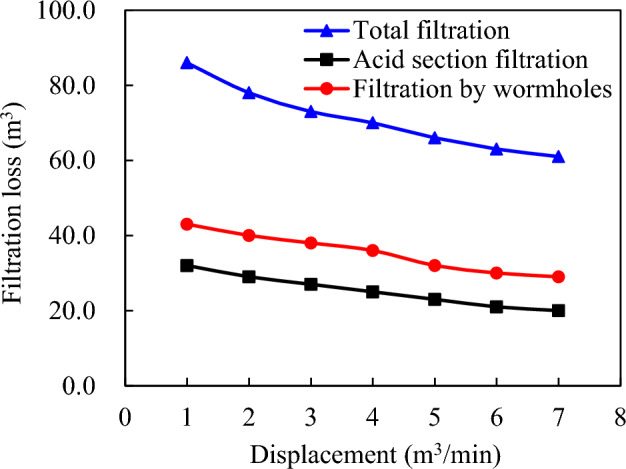
Composition of total acid fluid filtration loss under different pumping displacement.

Furthermore, Fig. 13 illustrates that with the increase in pumping displacement, the acid fluid loss decreases. This occurs despite the fact that an increase in pumping displacement leads to an increase in net fracture pressure. This higher pressure, in turn, leads to an increase in acid fluid wall filtration and wormhole filtration. However, this increase in pumping displacement also reduces the construction time. This shorter construction period not only shortens the acid fluid filtration time but also reduces the overall acid fluid filtration. This suggests that while pursuing a higher pumping displacement may offer some benefits in terms of acid fluid loss reduction, it may not necessarily result in an increased acid etching distance.

#### Acid consumption

**Figure 14:**
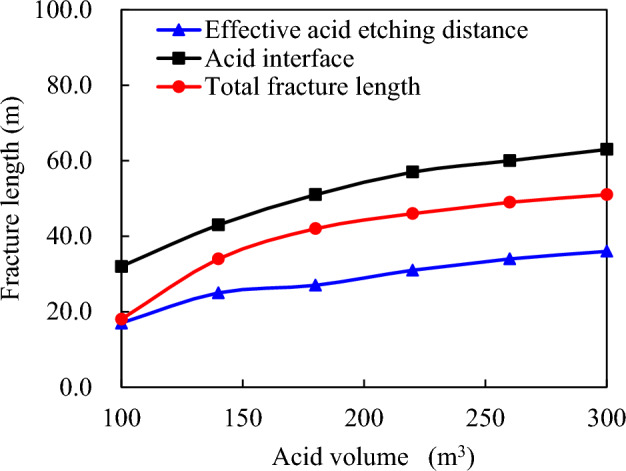
Effect of acid consumption on acid etching distance.

With an acid viscosity of 10 mPa·s, acid concentration of 20%, and pumping displacement of 4.0 m^3^/min, the acid scales at 100.0, 140.0, 180.0, 220.0, 260.0, and 300.0 m^3^ are illustrated in Fig. 14 to demonstrate the relationship between acid consumption and its etching effectiveness. The effective distance of acid etching progressively extends as the consumption increases from 100.0 to 300.0 m^3^, indicating that higher acid consumption results in a more profound etching effect; however, beyond 260.0 m^3^, further increases do not yield notable benefits. The total length of the acid liquid interface and acid fracturing fracture also exhibit interesting trends with respect to acid consumption. As the consumption increases, so does the total length of both interfaces, suggesting that a higher acid concentration results in a more extensive interaction between the acid and the material being etched, leading to a longer etching distance. However, after surpassing 260.0 m^3^, the increment in the total length of these interfaces begins to slow down, which could be an indication that the maximum potential of the acid has been reached, and further increases in acid consumption become less effective in extending the etching distance. In conclusion, the data presented in Fig. 14 provide valuable insights into the correlation between acid consumption, etching distance, and the efficacy of acid etching processes. While increased acid consumption generally results in a greater etching distance, there exists a threshold beyond which further increases in acid consumption yield diminishing returns.

**Figure 15:**
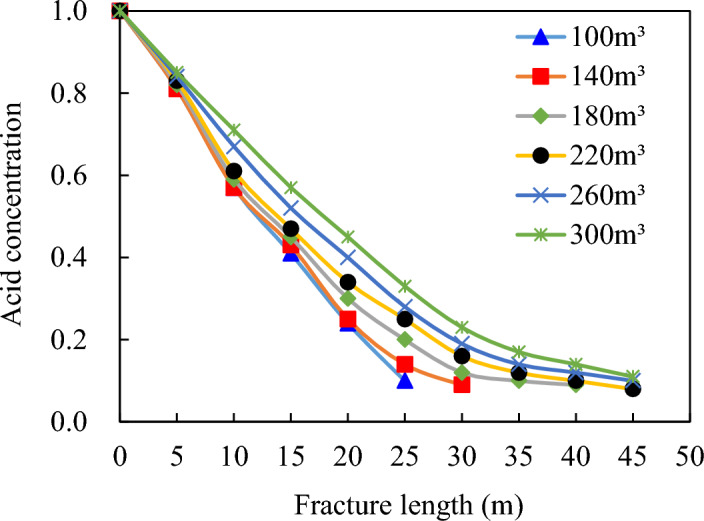
Distribution of acid concentration in fracture under different acid amounts.

As the volume of acid used in the acid-rock reaction increases, the reaction process is gradually supplemented by the acid solution. This leads to continuous expansion of the acid-etching wormholes (Fig. 15). Consequently, the filtering effect produced by these acid-etching wormholes significantly intensifies. It may appear that a higher volume of acid would result in a longer effective acid etching fracture length.

**Figure 16:**
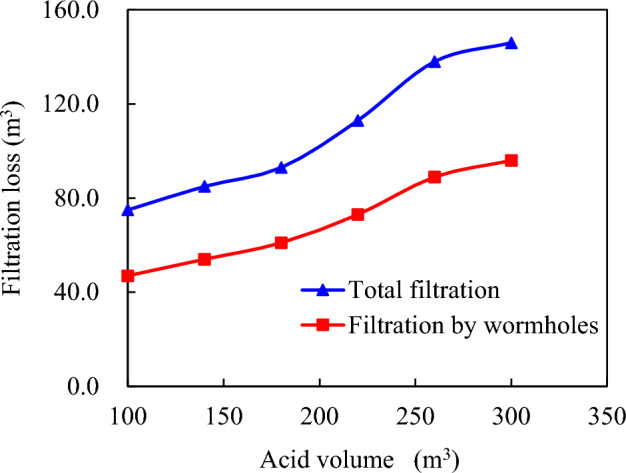
Composition of total filtration loss of acid solution under different acid solution dosage.

However, once the amount of acid used exceeds 260.0 m^3^, the rate of the acid-rock reaction begins to slow down. This deceleration is due to the interaction between ionic effects and the diffusion rate of acid within the wormholes, as depicted in Fig. 16. The gradual increase in filtration of acidic liquor visually represents this slowing reaction rate. In other words, increasing the volume of acid does not guarantee a proportional extension of effective acid etching fracture length; instead, most of the acid is likely lost through wormhole filtration processes, emphasizing the importance of maintaining a balance between acidic volume and reaction rate for optimizing an efficient and desired etching outcome.

### The influence of acid etching fracture parameters

#### Acid etching fracture width

**Figure 17:**
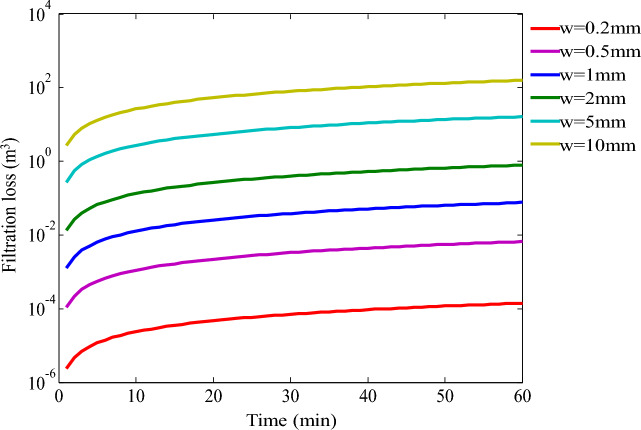
Variation of acid filtrate loss with time under different acid etching fracture widths.

Assuming all other influencing factors remain consistent, we simulated the filtration process of a 20% acid solution through the crevice in the fracture wall for various acid etching fracture widths, including 0.1 mm, 0.5 mm, 1.0 mm, 2.0 mm, 5.0 mm, and 10.0 mm (Fig. 17). The simulation results demonstrate that the cumulative filtration rate of the 20% acid solution increases in correspondence with the expansion of the acid-etched fracture width. There is a discernible peak value in the filtration rate during the initial stages of acid penetration into the fracture, indicating a sudden and pronounced acceleration in the filtration process followed by a relatively gentle increase suggesting a gradual yet consistent acceleration which ultimately stabilizes at a continuously escalating rate.

#### Distribution of acid concentration in acid etched fracture

**Figure 18:**
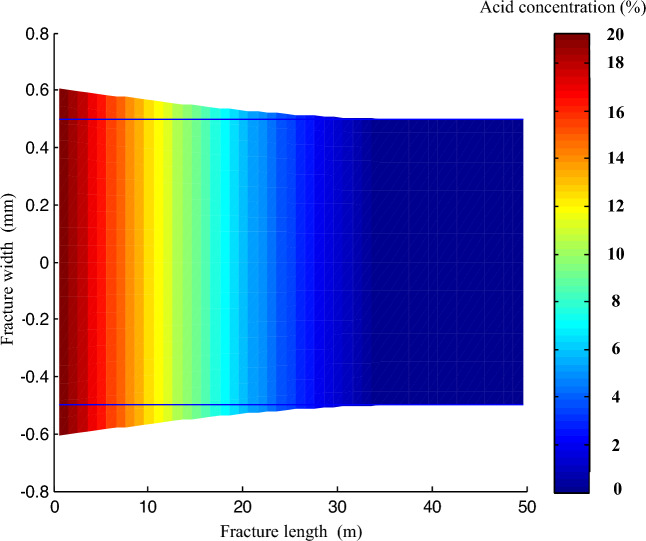
Plan of acid concentration distribution inside acid etched fracture.

The simulation meticulously analyzed the concentration distribution of a 20% acid solution within a two-dimensional acid etching fracture, specifically focusing on its width of 1.2 mm. The dynamic variation of the acid concentration was also studied to understand how it changes as the fracture extends. As shown in Fig. 18, the acid concentration peaks at the open end of the fracture due to its reaction with the rock on the fracture wall, leading to a gradual decrease in concentration as the fracture extends. The simulation revealed that this decreasing trend eventually results in an acid concentration of 0 at the apex of the fracture.

**Figure 19:**
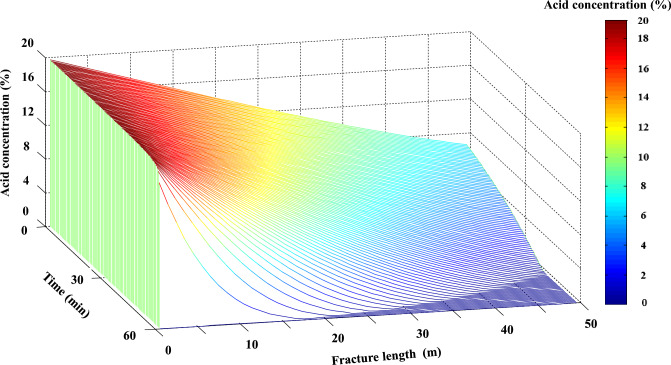
Three-dimensional dynamic map of acid concentration distribution inside acid etched fracture.

In consideration of the ongoing expansion and propagation of acid etching fractures during practical acidizing operations, we conducted a simulation to analyze the concentration distribution of 20% acid within three-dimensional acid etching fractures. Our analysis also took into account the interaction between the acid and the fracture wall, as well as the presence of acid filtration (Fig. 19). The gradual reduction in acid concentration along the direction of fracture extension during the process of acidizing enables us to determine the distribution of acid concentration within the fractures at any given time during construction, thereby facilitating optimization of parameters for designing effective acidizing processes.

### Model validation and analysis

The gas well was chosen and simulated using both the paper model and a commercial acid injection software. Comparative analysis of the results indicates that the acid erosion fracture parameters calculated by the paper model closely align with those obtained from the commercial software, exhibiting an error range of less than 10%. However, it is observed that at the fracture tip, the acid concentration estimated by the paper model surpasses that derived from the commercial software (Table 1). This discrepancy may arise due to neglecting acid filtration caused by natural fractures in the thesis model, leading to a higher concentration estimation. Nevertheless, this deviation remains within manageable limits.

**Table 1:** Comparative analysis of simulation results between the theoretical model and acid fracturing software.

Calculation results	Acid etching fracture length/m	Average fracture width/mm	Acid concentration at the fracture tip/%
Method			
Calculation results of the paper model	58	6.83	8.27
Commercial acid pressing software calculation results	53	7.36	4.81

## Conclusions

1. The incorporation of acid-etching wormhole filtration enhances the precision of fluid dynamic filtration simulation in acid fracturing. Taking into account the coefficient for acid etching filtration significantly increases the calculated distance compared to relying solely on wormhole filtration in acid fracturing. Field tests and analysis have demonstrated that integrating calculations for acidic erosion-induced wormhole filtrations results in a substantially greater effective distance, validating its inclusion in our study on acid fracturing.
2. The concentration of acid within the range of 15% to 20% exerts the most significant influence on the effectiveness of acid fracturing, while concentrations below 15% and above 20% have a negligible impact. Acid pumping displacement plays a crucial role; however, when it exceeds a threshold of more than 5.0 m3/min, the rate of acid etching slows down as pumping displacement increases. Therefore, optimizing pumping displacement is essential when selecting construction parameters to avoid unnecessary vehicle fracturing and minimize costs.
3. The viscosity of the fluid significantly impacts acid fracturing construction. Low-viscosity fluids lead to increased filtration and expansion of acid-etching wormholes, while high-viscosity liquids impede flow properties and reduce filtration. By integrating modified modeling techniques to quantify acid-rock reactions in these fractures, it accurately assesses their extent and optimizes design parameters for effective acidification. This approach ensures maximum efficiency with limited amounts of acid, reducing costs and improving operational effectiveness in oil and gas field development.

## Data Availability

The datasets used and/or analyzed during the current study available from the corresponding author on reasonable request.
